# The Transmembrane Domain and Acidic Lipid Flip-Flop Regulates Voltage-Dependent Fusion Mediated by Class II and III Viral Proteins

**DOI:** 10.1371/journal.pone.0076174

**Published:** 2013-10-04

**Authors:** Ruben M. Markosyan, Fredric S. Cohen

**Affiliations:** Department of Molecular Biophysics and Physiology, Rush University Medical Center, Chicago, Illinois, United States of America; University of Illinois at Chicago, United States of America

## Abstract

Voltage dependence of fusion induced by class II and class III viral fusion proteins was investigated. Class II proteins from Ross River and Sindbus virus and a mutant class III protein from Epstein Barr virus were found to induce cell-cell fusion that is voltage dependent. Combined with previous studies, in all, four class II and two class III protein have now been shown to exhibit voltage-dependent fusion, demonstrating that this is probably a general phenomenon for these two classes of viral fusion proteins. In the present study, monitoring fusion of pseudovirus expressing Vesicular Stomatitis virus (VSV) G within endosomes shows that here, too, fusion is voltage dependent. This supports the claim that voltage dependence of fusion is biologically relevant and that cell-cell fusion reliably models the voltage dependence. Fusion induced by class I viral proteins is independent of voltage; chimeras expressing the ectodomain of a class I fusion protein and the transmembrane domain of VSV G could therefore be used to explore the location within the protein responsible for voltage dependence. Results showed that the transmembrane domain is the region associated with voltage dependence. Experiments in which cells were enriched with acidic lipids led to the conclusion that it is the flip-flop of acidic lipids that carries the charge responsible for the observed voltage dependence of fusion. This flip-flop occurred downstream of hemifusion, in accord with previous findings that the voltage dependent steps of fusion occur at a stage subsequent to hemifusion.

## Introduction

The structures of the viral fusion proteins that mediate membrane fusion, as identified crystallographically, fall into three different classes [1]. All viruses that contain class II or class III fusion proteins initiate infection by fusing within endosomes. Some viruses containing class I fusion proteins also fuse within endosomes (e.g., influenza), while others (e.g., measles) fuse to plasma membranes.

It is experimentally possible to study fusion within endosomes [2], [3], [4], but the interiors of these organelles are difficult to access. Consequently, fusion within endosomes is commonly modeled by binding virus or cells expressing fusion proteins to target cells, and then lowering the pH of the external solutions. This procedure mimics endosomal low pH-triggering functions. We have previously reported that the extents of membrane fusion to cell membranes–mediated by the class II proteins Semliki Forest virus (SFV) E1/E2 and Venezuelan Equine Encephalitis virus (VEEV) E and the class III protein Vesicular Stomatitis virus (VSV) G–vary with the voltage across the plasma membrane of the target cell [5], [6]. Fusion is much more likely to occur when the intracellular voltage is negative with respect to the extracellular solution (as occurs naturally) than when voltage is positive. In contrast, for the class I proteins we have tested, fusion proceeds independent of voltage for both plasma membrane and endosomal cases. We found that for the class II and III proteins, voltage has no affect on the process of hemifusion, but voltage does control steps downstream of hemifusion that lead to pore formation. Therefore, the sensor of membrane voltage–wherever it is located on the protein–must be inserted within the target membrane subsequent to hemifusion.

In this study, we investigated four central aspects of voltage-dependent fusion. (1) Is dependence of fusion on voltage a universal property of class II and III proteins? We tested this by measuring fusion induced by two additional class II proteins and one additional class III protein. Fusion induced by each of the three proteins was found to be voltage dependent. (2) Is fusion of virus within endosomes, the biologically relevant location for infection, also voltage-dependent? We measured extents of fusion of pseudovirus within endosomes and found that it was voltage dependent and qualitatively similar to extents of fusion to plasma membranes. (3) Which region of class II and III proteins confers voltage dependence? As part of creating a fusion pore, the transmembrane domain (TMD) inserts into the target membrane subsequent to hemifusion [7]; we found that it is the segment that confers voltage dependence. (4) What molecular moieties could be carrying the electrical charges that must move through membranes to effect voltage-dependent fusion? Although the TMD is the region of the protein that determines whether fusion is voltage dependent, the charge movement need not reside within this structure. Because the headgroups of acidic (i.e., negatively charged) phospholipids can respond to voltage, their voltage driven flip-flop across the target membrane could be the relevant charge movement. We found that this is indeed the case.

## Materials and Methods

### Cells, Pseudovirus, and Reagents

For cell-cell fusion experiments, the effector/target cell pair combinations for class I fusion proteins were: HAb2 cells (provided by Judy White, University of Virginia) stably expressing influenza hemaglutinin (HA)/HEK 293 T cells [8]; NIH 3T3 cells [9] transfected to express ASLV Env/HEK293T cells expressing the receptor TVa [10]; HEK 293T cells expressing JRSV Env (or truncated mutants)/HAb2 cells; TF228.1.16 cells [11] stably expressing HIV-1 Env [11]/Hela T4+ cells [12]. For class II or III viral fusion proteins, HEK 293T cells were transfected with a plasmid to express the indicated protein and HAb2 cells were used as their targets. Chimeras with HA as ectodomain and VSV G as TMD were expressed in HEK 293T cells by transfection and fused to Cos7 cells [13] as targets. The plasmids were obtained from: Douglas Lyles (Wake Forest University) for VSV G; Shan-Lu Liu (University of Missouri) for JRSV Env and truncated TMD mutants; Margaret Killian for SFV E1/E2 and chimeras (Albert Einstein College of Medicine; Richard Kuhn (Purdue University) for Ross River virus E and Sindbis virus E1/E2; Richard Longdecker (Northwestern School of Medicine) for EBV gB801; John Young (Salk Institute) for ASLV Env.

For experiments in which NBD-PG (Avanti Polar Lipids, Alabaster, AL) was incorporated into either HAb2 cells or HEK 293T cells expressing VSV G, a stock 2.5 mM solution in ethanol was added at a dilution to yield the indicated concentrations and incubated at 4°C for 5 min. Unincorporated fluorescent lipid was eliminated by spinning down the cells. For reversibility experiments, NBD-PG was removed from membranes by incubating cells for 5 min at 4°C in a phosphate-buffered saline containing 10 mg/ml delipidated BSA and washing with fresh solution. The washing procedure was repeated a second time. Fluorescence microscopy was used to confirm that the fluorescent lipid had, indeed, been removed.

Pseudovirus was prepared as previously described [5], using an SFV E1/E2 or VSV G plasmid, as indicated, to express the desired fusion protein in the viral envelope. The viral envelope contained the fluorescent lipid dye DiD and the core was labeled by GFP at the C-terminus of the nucleocapsid protein (NC-GFP) of MLV. For fusion experiments, cells were adhered to polylysine-coated cover slips and pseudovirions were bound to the tops of the cells through spinoculation. Virus within endosomes was located by confocal microscopy, and internalized virions that had lost their NC-GFP were scored as fusion.

### Measuring Extents of Cell-cell Fusion

Effector and target cells were mixed together in a small tube and then plated onto polylysine-treated glass coverslips. After a 30 min incubation at room temperature, pH was lowered for 10 min, the solution reneutralized, and temperature then raised to 37°C for 30 min. In the case of effector cells expressing HIV Env as the fusion protein, low pH is not required for fusion and so effector and target cells were incubated at 23°C for 2.5 hrs at neutral pH, and then temperature was raised to 37°C for 30 min. To measure fusion at positive voltages, 30 µM SQI-Pr was maintained in solution at all steps of the procedure for all cell types. Effector cells were loaded with 1.3 µM calcein AM (Invitrogen) and target cells were loaded with 30 µM CMAC (Invitrogen). For experiments in which NBD-PG or NBD-PC were incorporated in cell membranes, effector cells were loaded with 1.0 µM (5-(and-6)-(((4-Chloromethyl)Benzoyl)Amino)Tetramethylrhodamine; CMTMR, Invitrogen), and target cells were loaded with 1.3 µM calcein-AM. Fusion was quantified off-line using video microscopy to identify the spread of these dyes for effector/target cell pairs.

The intermediate of CAS was generated by incubating bound effector and target cells at 4°C for 30 min, then lowering pH for 10 min at 4°C, followed by reneutralization, also at 4°C. Fusion was induced by raising temperature to 37°C after the cells were maintained at neutral pH for 10 min [6]. For experiments in which NBD-PG was removed from cell membranes, the washing procedure described above was used.

### Controlling Voltage

For electrical control of voltages, the target cell (bound to an effector cell) was placed in whole-cell patch-clamp mode, capacitance measurements were performed, and pore conductance was calculated as previously described [14]. The solution placed inside the patch pipette consisted of 135 mM cesium glutamate-5 mM MgCl_2_-5 mM BAPTA [1,2-bis(*o*-aminophenoxy)ethane-*N*,*N*,*N*′,*N*′-tetraacetate]-10 mM HEPES (pH 7.2); the external solution was 135 mM *N*-methyl-glucamine aspartate-5 mM MgCl_2_-2 mM HEPES (pH 7.2). For ionophore experiments, 30 µM of the sodium ionophore SQI-Pr (TefLabs, Austin, TX) was added to the solution containing bound effector and target cells. Whole cell patch clamp experiments confirmed that for a standard external solution containing 135–140 mM Na^+^, the ionophore shifted intracellular voltages from negative to positive.

## Results

### 1. All Class II and III Proteins Tested Exhibit Voltage-dependent Fusion, whereas All Class I Proteins Tested Exhibit Voltage-independent Fusion

We used ionophore experiments to measure cell-cell fusion mediated by four class I proteins (Influenza HA, Avian Sarcosis Leukemina virus (ASLV) Env, Jaagsiekte sheep retrovirus (JRSV) Env, and Human Immunodeficiency virus (HIV) Env), three class II proteins (Semliki Forest virus E1, E2, Ross River Virus (RRV) E1/E2 and Sindbus virus E1/E2), and by two class III proteins, (VSV G and Epstein Barr Virus (EBV)). Herpes viruses, including EBV, require several different viral proteins (gB, gH, and gL) for fusion to occur [15]. It was not certain which of these proteins were fusogens, and which served accessory roles, but a mutant form of gB (referred to as gB801 [15]–a highly conserved protein within the herpes virus family–was generated that expressed well on cell surfaces and induced syncytia formation without the need of other viral proteins [15], suggesting that gB is the primary fusion protein and the others (gH and gL) are accessory proteins. We used this mutant gB801 for EBV experiments, and, consistent with this suggestion, found that alone, it yields very high levels of fusion.

**Figure 1:**
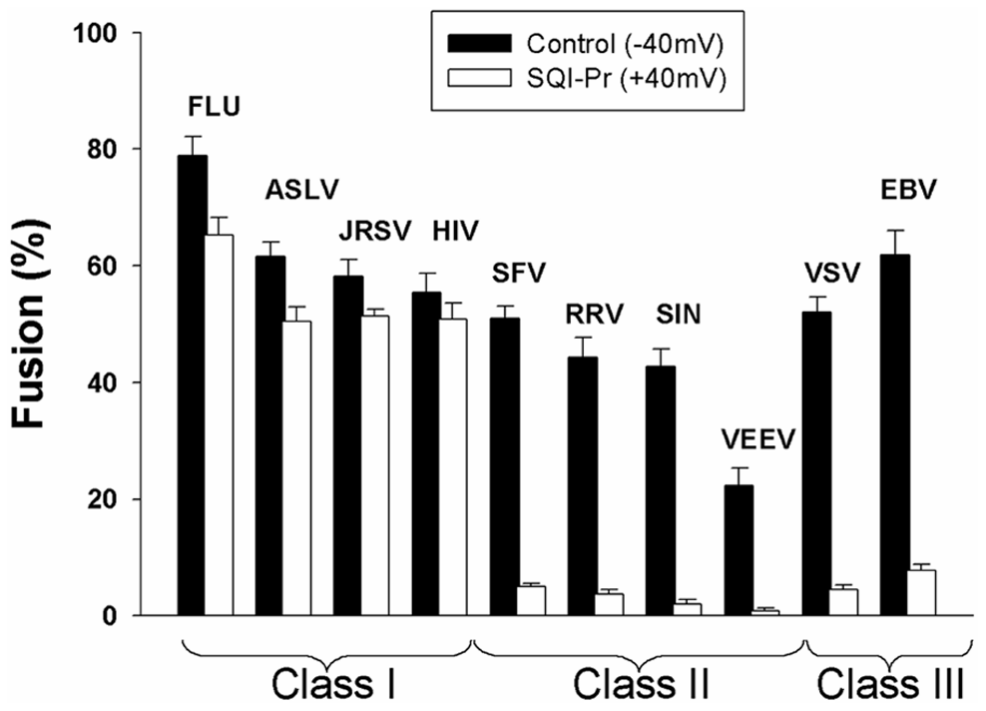
Fusion induced by class II and III fusion proteins is voltage dependent. Extents of cell-cell fusion induced by fusion proteins from the three viral classes. The fusion proteins were: class I–Influenza HA (Flu), ASLV Env, JRSV Env, and HIV Env; class II–SFV E1/E2, RRV E1/E2, Sindbis E1/E2, VEEV E; class III–VSV G and a mutant (gB801) of EBV (class III). Fusion was independent of voltage for the four class I proteins tested. In contrast, fusion was significantly greater at negative (filled bars) than positive potentials (open bars) for all class II and III proteins. Effector cells were transfected with the proteins of the indicated viral types. Voltage was made positive by adding the Na^+^ ionophore SQI-Pr (30 µM). Error bars are SEM (n = 3).

We used a standard cell-cell fusion system to investigate voltage requirements of the tested proteins to induce fusion [6]. Transfected effector cells that express the fusion protein were bound to target cells, and the external solutions were acidified. Spread of fluorescent aqueous dye between bound effector and target cells was used to report fusion. In order to significantly alter the natural negative potential across plasma membranes of large numbers of bound cell pairs, we added the Na^+^-ionophore SQI-Pr (30 µM) to the high Na^+^-containing external solution. By electrically measuring the voltage across the plasma membranes of target cells, we verified that the ionophore altered membrane potentials. The resting potentials of the cells were naturally ∼ −40 to –50 mV and were shifted to ∼ +35 mV by addition of the ionophore. As we found earlier by electrical capacitance measurements [6], the extents of fusion induced by class I proteins were independent of voltage for ionophore control of membrane potentials (Fig. 1, ∼1500 cell pairs assayed for each column). In contrast, the spread of aqueous fluorescent dye between effector and target cells showed that fusion was significantly reduced by the ionophore for each of the class II and III proteins tested (class I: SFV E1/E2, RRV E1/E2, Sin E1/E2, VEEV E; class III: VSV G, EBV gB801). This regulation of cell-cell fusion by voltage occurred in the same manner as we reported for the class II and III proteins (SFV E1/E2, VEEV E, and VSV G) tested earlier by capacitance measurements [6]. These new experiments extend our observation that the dependence of fusion on voltage is based on the class of the fusion protein.

### 2. Endosomal Fusion is Blocked by Positive Potentials

**Figure 2:**
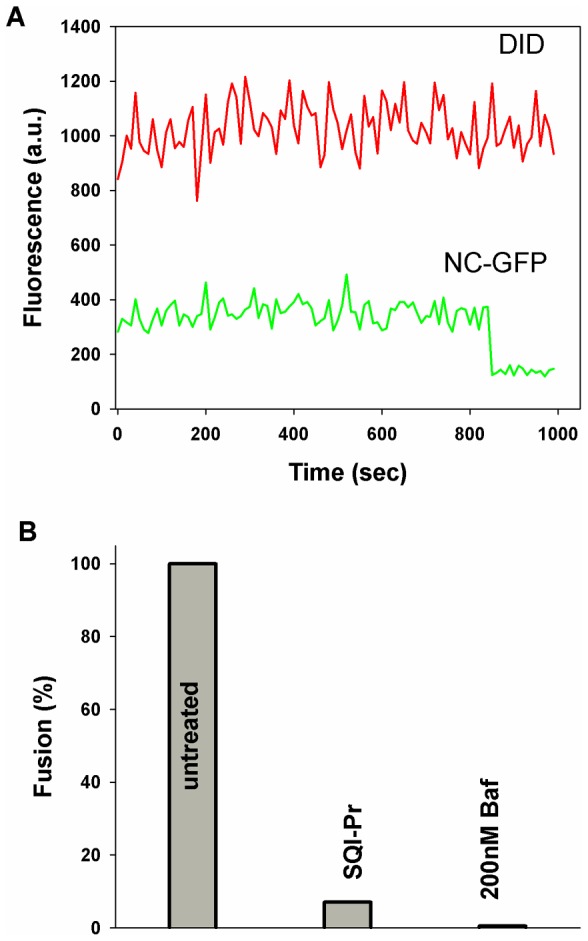
Fusion of virus within endosomes is voltage dependent. A. Fluorescence profiles of viral fusion within endosomes are distinct from fusion to plasma membranes. A typical record shows that the observed fluorescence of lipid dye (DiD) within a viral envelope does not change upon fusion to endosomes, which are submicroscopic. The NC-GFP within the viral core is released after fusion, resulting in decreased fluorescence. B. Fusion within endosomes of pseudovirus bearing SFV E1/E2 was quantified for untreated CV-1 cells (first column). In experiments performed with the same protocol, except that SQI-Pr (30 µM) was added to make endosomal potentials positive, extents of fusion were low (second column). In experiments in which bafilomycin A1 (200 nM was added instead of SQI-Pr, to neutralize the interior of endosomes, fusion was completely eliminated (third column).

We tested whether the observed voltage dependence of fusion to plasma membranes is also true in the native environment of endosomes. We created pseudovirus expressing VSV G as the fusion protein, with envelopes labeled with the fluorescent lipid analog DiD and cores marked by GFP [5]. CV-1 cells were grown overnight on untreated cover slips and pseudovirus were then bound on top of the cells by centrifugation at 4°C for 1 hr (i.e., by spinoculation). We then raised temperature to 37°C, and waited 15 min to allow natural internalization of pseudovirus, recorded continuously, and analyzed the data off-line. When fusion occurs within an endosome, DiD fluorescence is maintained, because the diameter of the endosome is less than optical resolution. The GFP fluorescence, however, disappears (Fig. 2A, typical record). This pattern of fluorescence is distinct from that of fusion of virus to plasma membranes in which both dyes disappear. Virus bound to the plasma membrane is not exposed to low pH and thus cannot fuse.

Of the 1420 virions that were initially bound to the plasma membrane, we found that ∼4% of them fused from within endosomes (Fig. 2B, first bar). In control experiments, we added bafilomycin A (200 nM) to prevent acidification of endosomes [16]. Here, 750 virions were initially bound to the plasma membranes; not a single fusion event was observed (third bar  = 0). This demonstrates that the fusion events we counted indeed occurred within low-pH endosomes. We switched the potential across the endosomal membranes from negative to positive by adding the Na^+^-ionophore (30 µM SQI-Pr, second bar). For 1150 bound virions, only 0.25% fused, showing that the ionophore abolished fusion by more than an order of magnitude. This reduction in fusion is in accord with our measured voltage dependence [5], the literature values of endosomal voltage [17], and our electrically determined estimate for voltage in the presence of the ionophore. Clearly, fusion within endosomes induced by VSV G is severely reduced through switching of membrane potentials to positive values.

### 3. Voltage Dependent Fusion is Conferred through the TMD

**Figure 3:**
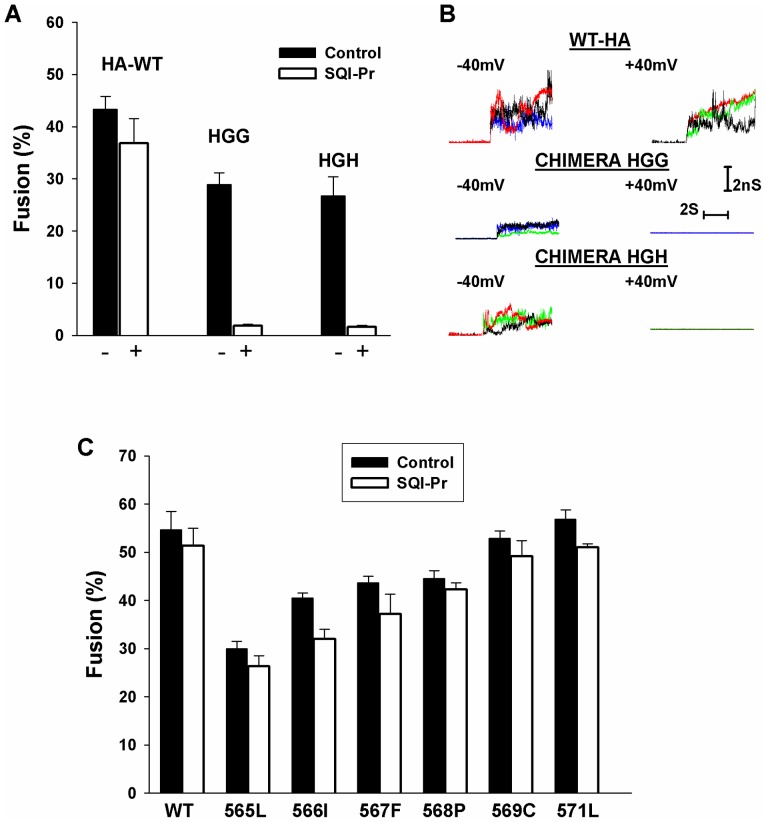
Voltage dependence is determined by the identity of the TMD. A. Cell-cell fusion induced by WT-HA was independent of voltage (first columns). But swapping the TMD of VSV G for that of HA resulted in fusion that was much greater for negative (filled bars) voltages than for positive (open bars) voltages (generated by the addition of SQI-Pr), independent of whether the CT was that of VSV G (second columns) or HA (third columns). Error bars are SEM (n  = 3). B. Electrical capacitance measurements show that fusion pores did not form at positive voltages across the target membrane when a chimera, containing a TMD derived from VSV G, was the fusion protein. The initial pore conductances and early patterns of growth (at negative voltages) do not depend on the identity of the TMD. The conductance profiles are not markedly different from those of WT influenza HA (top trace) when both the TMD and CT of VSV G are swapped for those of HA (middle traces), or just the TMD (bottom traces) is switched. The electrical capacitance measurements confirm the conclusion from ionophore experiments that the dependence of fusion on voltage follows the class of viral protein from which the TMD is derived. C. Reducing the length of the TMD of a class I fusion protein does not confer voltage dependence to fusion. The CT of the fusion protein of JRSV Env was eliminated and the TMD truncated one amino acid at a time. Truncation reduced the extents of fusion compared to WT, but fusion remained independent of voltage for all lengths of the TMD. The horizontal axis denotes the last (C-terminal) residue of each TMD truncation. Error bars are SEM (n ≥3).

To determine whether the TMD is the source of voltage-dependent fusion, we used chimeras consisting of the ectodomain of a class I fusion protein, the TMD of a class III protein, and the cytoplasmic tail (CT) of either the class I or class III protein. The chimeras were constructed from the ectodomain of influenza hemagglutinin (HA), the TMD of VSV G, and the CT of either VSV G (denoted HGG) or HA (denoted HGH). We have previously shown that cell-cell fusion induced by influenza HA is independent of voltage across the target membrane [6]. We transfected effector cells with these chimeras and bound them to target cells. We acidified external solutions to induce cell-cell fusion at either the natural negative voltage of the target cells (control, nominally −40 mV) or at +40 mV, established by adding the Na^+^ ionophore SQI-Pr (Fig. 3). Fusion was the same at +40 mV as at –40 mV for WT HA (Fig. 3A, first set of bars; voltage independence also shown in Fig. 1 and [6]). However, when TMDs of VSV G were swapped for those of HA, fusion became voltage dependent: fusion proceeded at −40 mV, but did not occur at +40 mV for either HGG (second set of bars) or HGH (third set of bars). This is the same dependence as was observed for fusion mediated by WT VSV G (Fig. 1 and [5]). To rigorously establish that voltage dependence is conferred by switching the TMD of influenza HA with that of VSV G, we performed voltage clamp capacitance experiments. We electrically clamped the target cell at −40 mV and observed, for HGG, fusion pores in 4 out of 12 experiments. When target cells were held at +40 mV, pores did not form in any of 10 experiments. Statistically, this would not occur if the probability for pore formation was the same for negative and positive voltages (p<0.05). We conclude that the TMD of VSV G confers voltage dependence. These capacitance measurements also showed that the fusion pores formed by the chimeras HGG and HGH at −40 mV had the same initial conductances and slow rates of pore growth as did pores formed by WT-HA (Fig. 3B, calculated conductance traces shown for several pores). Fusion pores were never observed at +40 mV for either of the chimeras (flat conductance traces); pores created by WT-HA at positive voltages were indistinguishable from those that formed at negative voltages.

TMDs of class II and III proteins are generally shorter than those of class I, and this may somehow be related to voltage dependence. The effects of truncation on voltage dependence were tested by expressing the class I protein JRSV Env and TMD-truncated mutants, and measuring voltage dependence of fusion to target cells. Based on hydrophobicity, the TMD of JRSV Env extends from residues numbers 550 to 571 [18]. Truncating the TMD (in addition to the CT) one amino acid at a time results in progressively less fusion (Fig. 3C). But the extent of fusion at negative voltages (filled bars) was always the same as that at positive voltages (open bars). Thus, the length of the TMD is not a critical factor for conferring voltage dependence to fusion.

### 4. Charge Movement Across Membranes is Due to Flip-flop of Acidic Lipids

Although only a small percentage of the acidic (negatively charged) membrane lipids reside within the outer (non-cytoplasmic) leaflet, it is known that for some class II and III fusion proteins, these acidic lipids are required for fusion to occur [19], [20]. The presence of acidic lipids as an aid in fusion may be a general phenomenon for class II and III proteins. This is difficult to test in a biological setting, however, since some acidic lipids are always present in outer leaflets. Flip-flop of acidic lipids by ATP-dependent translocases is likely to be inherently voltage dependent because the movement of each lipid’s negative charge across the entirety of the membrane should be affected by the membrane potential. It is thus possible that voltage-driven flip-flop is the underlying reason for voltage dependence of fusion. Under optimal fusion conditions (pH well below threshold and temperature at physiological 37°C), all fusion proteins undergo their low pH-induced conformational changes by the time of hemifusion [21], [22],[23]. For negative membrane potentials and natural lipid compositions, there is enough acidic lipid in the outer leaflets to promote the maximal extent of fusion that is possible. The amount of acidic lipids in outer leaflets would be reduced by flip-flop at positive potentials, and this would reduce the number of fusion events if acidic lipids are required: the extents of fusion would depend on voltage through lipid flip-flop. But the situation changes after experimentally enriching membranes with acidic lipids: The extents of fusion at negative voltages should not be augmented by acidic lipid enrichment, since it should already be at its maximum. Although the level of fusion is very low for natural lipid concentrations at positive potentials, enough acidic lipids would remain in outer leaflets after enrichment to support the maximum (or close to maximum) extents of fusion. Consequently, the extent of fusion at negative and positive potentials should be near maximal, and therefore roughly the same. That is, the observed voltage dependence of fusion should be reduced or even abolished if flip-flop of acidic lipids is somehow intimately coupled to fusion for class II and III proteins.

**Figure 4:**
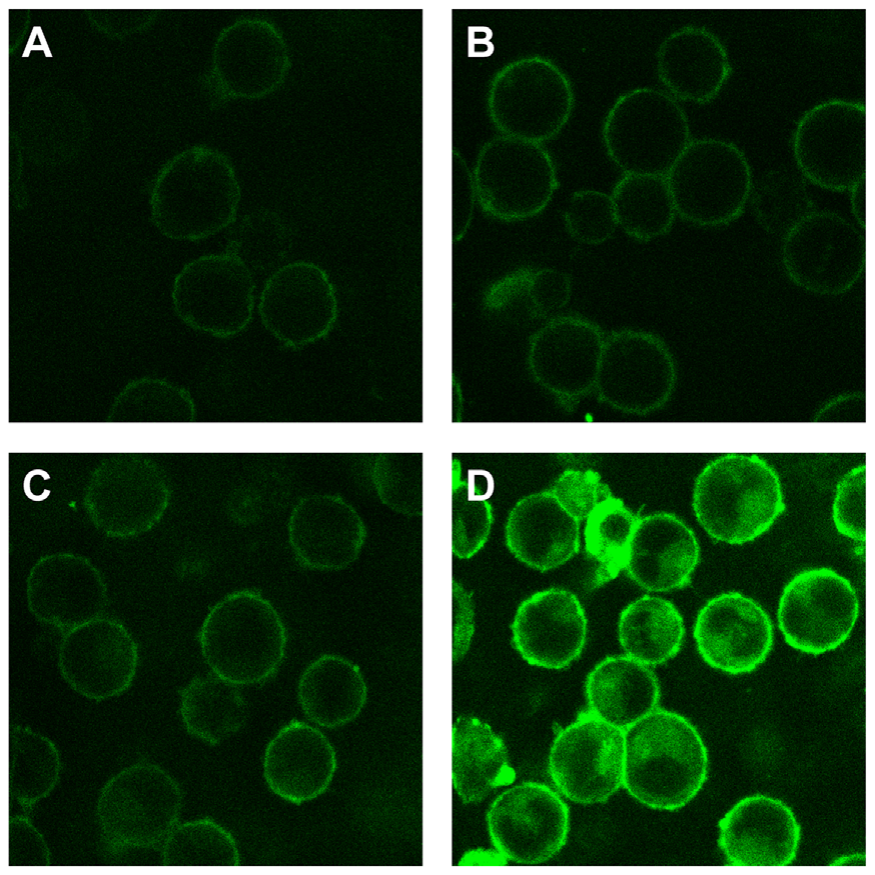
Cell populations are uniformly labeled by the addition of NBD-PG. As judged by fluorescence, NBD-PG incorporated to the same extent into almost all cells for each concentration of NBD-PG added to solution. The concentrations of NBD-PG were: (A) 0.025 µM; (B) 0.25 µM; (C) 2.5 µM; (D) 25 µM. (For fusion experiments, 2.5 µM NBD-PG was generally added).

**Figure 5:**
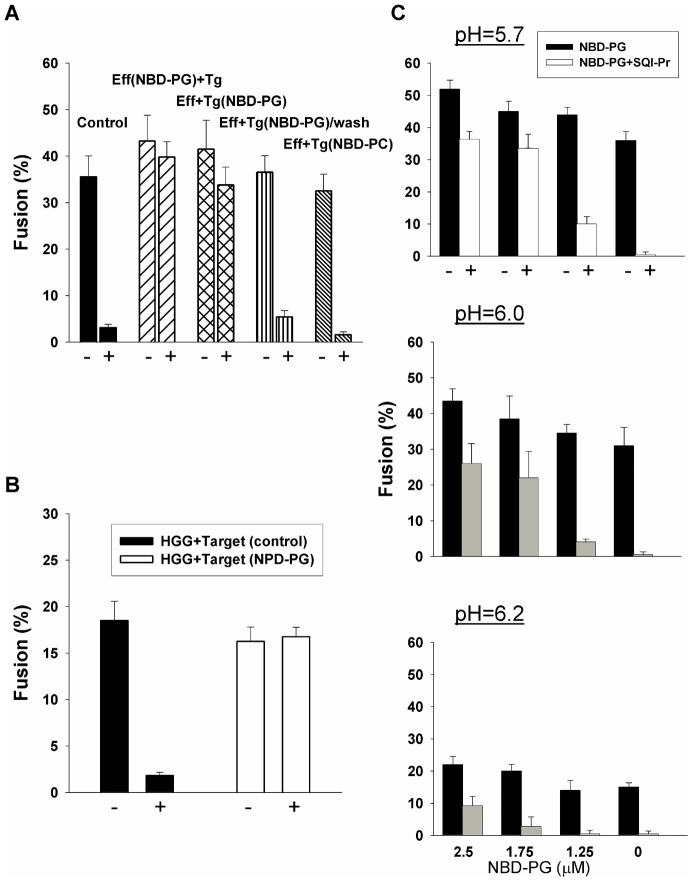
Presence of acidic lipids controls voltage dependency of fusion. A. The voltage-dependence of fusion induced by VSV G (first columns, solid bars) is virtually eliminated by incorporating NBD-PG into either the effector (second columns, hatched bars) or target cells (third columns, cross hatched bars). Washing out the NBD-PG (fourth columns, vertically lined bars) restored the voltage dependence of fusion. Voltages were made positive by the addition of SQI-Pr (30 µM). Addition of a neutral, zwitterionic lipid (NBD-PC) to cells does not affect voltage dependency of fusion (fifth columns, closely spaced hatched bars). B. The voltage dependent fusion induced by HA-G-G (first columns) was eliminated by the addition of an acidic lipid (NBD-PG). C. Increasing amounts of NBD-PG incorporated into membranes progressively reduces voltage dependence of fusion induced by VSV G. For all values of pH used to induce fusion, the extent of fusion at positive relative to negative voltages increases as more NBD-PG is allowed to incorporate into the membranes. Error bars are SEM (n ≥3).

We tested this flip-flop hypothesis by incorporating the acidic lipid PG (phosphatidylglycerol), fluorescently labeled by NBD, into either effector or target cells. PG is relatively water soluble for a lipid and this eases the task of introducing the lipid into extracellular solutions and removing it from membranes after it is incorporated. Fluorescence microscopy showed that all cells were highly fluorescent, establishing that high levels of PG were incorporated (Fig. 4). We expressed VSV G in effector cells and measured cell-cell fusion in the presence (positive membrane potential) and absence (negative potential) of SQI-Pr, for the same cells enriched or not enriched by NBD-PG. We carried out fusion experiments under optimal conditions. For cells in which NBD-PG was not incorporated (Fig. 5A, first set of bars), fusion was virtually abolished by the positive potential generated by SQI-Pr. But incorporating acidic lipids into either the effector (Eff bars) or the target cells (Tg bars) led to the elimination of the voltage dependence of fusion: the same extent of fusion was observed in the presence (+, positive potential) as in the absence of the ionophore (−, negative potential), as would be true if the flip-flop hypothesis is correct. Furthermore, the extent of fusion at negative potentials was not increased by incorporation of NBD-PG, also in agreement with our predictions. We removed NBD-PG from all membranes by washing with a solution containing delipidated BSA. The decrease in cell fluorescence to the base level confirmed that the fluorescent lipid was effectively removed (Fig. 5A, fourth set of bars. Voltage dependence was restored. This reversibility strongly supports the hypothesis that the absence of acidic lipids from outer membrane leaflets at positive potentials reduces fusion, thereby accounting for voltage-dependent fusion.

**Figure 6:**
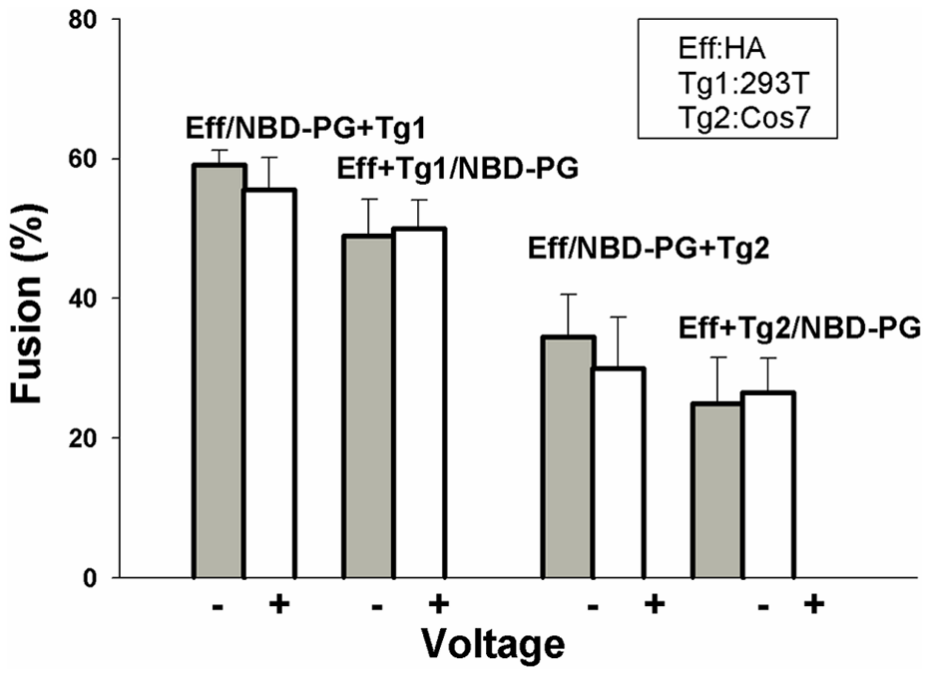
Enriching cells with NBD-PG does not alter the voltage dependence of fusion induced by influenza HA. HAb2 cells were used as effector (Eff) cells and either HEK 293T (Tg1, first two sets of columns) or Cos7 (Tg2, last two sets of columns) cells were used as targets. Regardless of target cell type, HA-mediated fusion remained independent of voltage after incorporating NBD-PG into either the effector (first and third set of columns) or target (second and fourth set of columns) cells. Voltage was switched from negative (shaded bars) to positive (open bars) by the addition of 30 µM SQI-Pr.

As a control, we incorporated NBD-PC, structurally identical to NBD-PG, except for its neutral, rather than negatively charged, headgroup. Identical experiments with cells enriched in NBD-PC were performed as described with NBD-PG. NBD-PC enrichment had no effect on voltage dependence (Fig. 5A, fifth set of bars). These results show that the concentration of acidic lipids has a direct effect on voltage-dependent fusion. As a further control, we used influenza HA as the fusion protein, incorporated NBD-PG into effector or target cells, and monitored fusion at negative and positive potentials. The incorporation of NBD-PG did not affect the lack of voltage-dependence fusion induced by the class I protein (Fig. 6).

Having established that voltage dependence of fusion was eliminated by acidic lipid enrichment, we tested whether the identity of the ectodomain was relevant to this elimination. We did this by using the HGG chimera for cell-cell fusion experiments in target cells enriched and not enriched in NBD-PG (Fig. 4B). The finding that the voltage dependence of fusion that occurs for natural, unriched target cells (first set of bars, control) is completely eliminated by incorporating the acidic lipid into target cells (second set of bars) clearly demonstrates that acidic lipids in combination with the TMD are somehow controlling whether fusion is voltage-dependent.

**Figure 7:**
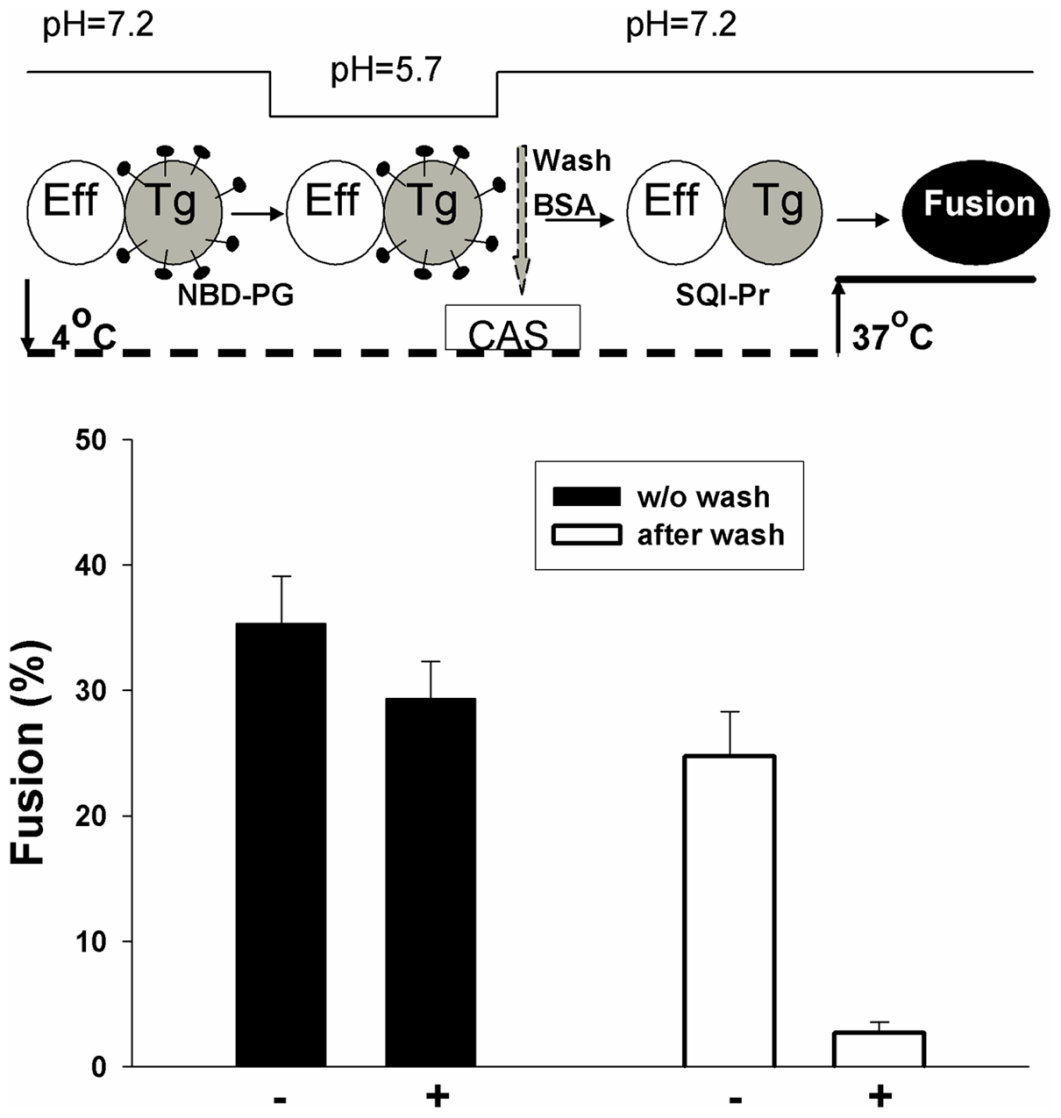
The reduction in voltage dependence caused by incorporating NBD-PG into membranes occurs downstream of hemifusion. Upper panel: Effector cells (open ellipse) expressing VSV G were bound, at 4°C, to target cells (filled ellipse) that were labeled with the lipid probe NBD-PG. A hemifusion intermediate (CAS) was created by acidifying at low temperature for 10 min, and the solution was then reneutralized. NBD-PG was either then removed by washing or allowed to remain. Fusion was induced by raising temperature to 37°C. Lower graph: When NBD-PG was present for the raised temperature, the extent of fusion was independent of voltage (first column, filled bars). When NBD-PG was removed after creating the hemifusion intermediate, fusion was voltage dependent (open bars). Therefore, NBD-PG acts downstream of hemifusion. Error bars are SEM (n ≥3).

Under suboptimal conditions, only a fraction of the fusion proteins will have undergone low-pH conformational changes at the point of hemifusion [22]. The density of activated fusion proteins can therefore by varied by changing the pH used to trigger fusion. We measured voltage dependence of fusion under suboptimal conditions by performing experiments at values of pH higher than those described thus far (pH 5.7) as a function of the concentration of added NBD-PG. The presence of NBD-PG still reduced voltage dependence but as pH was raised, the fractional reduction was less: for the highest concentration of NBD-PG, the relative reduction in fusion for positive voltages was greatest at pH 5.7, smallest at pH 6.2, and intermediate at pH 6.0. To locate the stage in the fusion process at which acidic lipids act, we enriched target cells with NBD-PG and acidified the aqueous solution to pH 5.7 at 4°C. Solutions were then reneutralized, yielding a cold-arrested state (CAS) (Fig. 7, upper panel schematically illustrates the protocol) that is known to be a state of hemifusion [6]. The temperature was then raised to 37°C either in the presence of absence of SQI-Pr. The resulting extents of fusion were independent of membrane potential (Fig. 7, first two columns). If, however, NBD-PG was removed at CAS by washing, the extent of fusion was voltage dependent (second two columns). It follows that the acidic lipid NBD-PG abolished the voltage dependence of fusion at a step downstream of hemifusion.

## Discussion

From the present and prior studies [6], we have to date tested four class II and two class III viral fusion proteins, and found that the fusion induced by each of them is voltage dependent. It is thus highly probable that the dependence of fusion on voltage is a general feature of class II and class III viral fusion proteins. In contrast, fusion induced by the four class I proteins we have tested did not show any dependency on voltage. Using SFV E1/E2 as the fusion protein, we found that voltage is a consequential regulator of viral fusion within endosomes. To test whether the TMD is the critical region of a fusion protein that determines the voltage dependency of fusion, and to ascertain whether charge movement of voltage dependence is due to flip-flop of acidic lipids, we used VSV G as our model protein. Our findings for SFV E1/E2 and VSV G provide a conceptual foundation for testing whether the mechanistic aspects of these two proteins generalize to other class II and III viral fusion proteins.

### gB is the Primary Fusion Protein of EBV

The finding that EBV gB801, a mutated form of WT gB, bypasses the need for auxilliary proteins for fusion to occur has precedents for other viral fusion proteins, notably HIV Env. HIV Env normally binds to CD4 on target T-cells and macrophages, resulting in conformational changes of the fusion protein that allow it to then engage chemokine receptors, whereupon it undergoes further conformational changes that culminate in fusion. But altered forms of HIV (and SIV) Env have been identified that do not require the presence of CD4 for fusion to proceed [24]. Evidence indicates that these naturally occurring mutated forms of HIV Env have altered conformations, allowing them to directly engage chemokine receptors [24], [25]. We propose that the multiple mutations that give rise to EBV gB801 allow the altered protein to reconfigure so that pH alone can trigger fusion.

### Endosomal Factors in Control of Viral Fusion

Since its discovery as the trigger within endosomes [26], [27], low pH has become almost the sole experimental tool, and the conceptual focus, in the study of cellular control of fusion between virus and an endosomal membrane. Combining the method of simulating the internal environment of endosomes by acidification of external solutions with various methods to measure fusion of cells that express viral proteins (or virus) to target cells is the most common experimental strategy today.

The sign of the potential across an endosomal membrane (negative within cytosol) is due to pumping of H^+^ into the endosome; the large potential generated by the pump is estimated to be reduced to ∼ −20 mV by Cl^−^ permeabilities [17]. Because electrical potential invariably controls important functions across any organelle membrane for which voltage is not zero, it is extremely likely that an endosomal voltage has functional consequences as well. We have shown that fusion mediated by class II proteins and class III proteins, triggered by acidification of external solutions, is dependent on the voltage across the target cell membrane: low pH triggers the conformational changes of the viral protein that allow portions of the protein to insert into the target membrane; but once insertion has occurred, voltage controls further movements of parts of the protein. The voltage across the endosomal membrane is thereby employed in the initiation of viral infection. Experimentally, the use of low pH alone to trigger fusion has obscured the role of additional factors in viral infection, and not taking them into account may cause experimental data to be misinterpreted to some degree. Because fusion mediated by class II and III viral proteins is facilitated by negative voltages–as occur naturally across endosome membranes–and is inhibited by positive voltages [5], [6], it is possible that if voltage could be interfered with, infection could be controlled.

### The Importance of TMDs in Voltage Dependence

Results from our chimera experiments indicate that the TMD is the structure that leads to voltage sensitivity Although possible, it is unlikely that these chimeras have misfolded or that their ectodomains have significantly different structures than those of WT influenza HA: Membrane fusion was reasonably efficient and did not exhibit any anomalies we could detect. Several other chimeras of ectodomains/TMDs of viral fusion proteins have been generated, either using TMDs from fusion proteins or from proteins unrelated to fusion [28],[29],[30],[31]. These chimeras induced fusion with patterns virtually identical to those of the ectodomain; any differences were related to initial pore conductance and other subtle parameters [28]. The fact that fusion and its characteristics have proved robust, rather than altered by a non-native TMD strongly indicates that the structural features required for a viral protein ectodomain to induce fusion are not dependent on the specific identity of the TMD.

Only membrane-inserted segments of a protein can respond to voltage. The TMDs, fusion loops, and other loops that might insert into the target membrane were thus all candidates for conferring voltage sensitivity. Because the voltage-dependent steps reside downstream of hemifusion, the stretch of amino acids, wherever it is located, should insert into the target membrane after hemifusion has been achieved. Fusion and other loops are thought to insert into target membranes as an early step in the fusion process [23], [32], integral to the creation of hemifusion; these loops play a central role in steps prior to voltage-dependent steps. The TMD does not play a prominent role in causing hemifusion [7], [33], [34]. It does not enter the target membrane until a hemifusion diaphragm has been created. After this hemifusion diaphragm has formed, the TMDs at the rim are thought to move into and through the diaphragm, thereby disrupting it to create a pore [7], [33], [35]. Our finding that voltage-dependent steps reside after hemifusion occurs is thus in full accord with the standard conception, which is based on both experiments [7], [34] and theory [36].

Because of the complexities of folding of class II and III fusion proteins into their native configurations, it may be difficult to swap a TMD of a class I protein for that of a class II or III protein, to determine whether the presence of a class I TMD eliminates voltage dependence of fusion. The feature that confers voltage-dependent fusion by TMDs of class II and III viral fusion proteins, but not class I proteins, now needs to be determined. All class II and III proteins are thought to arise by divergent evolution from their respective primordial proteins [37], [38]. Evolution from the same protein for each class provides a rationale for why TMDs of class II and III viral fusion proteins are shorter than those of class I proteins. More importantly, it appears that class II and III TMDs possess a conserved feature that confers voltage-dependent fusion. One possibility is that class II and class III TMDs couple to translocases for acidic lipids.

### The Role of Lipids in Voltage-dependent Fusion

In gating of voltage-dependent ion channels, the voltage sensor is encoded within the protein. One might therefore assume that in a voltage-dependent fusion process, the TMD of the fusion protein not only confers voltage dependence to fusion, but is itself also the voltage sensor. But lipids are directly involved in the membrane fusion process as two bilayers merge into one, and voltage can drive flip-flop of acidic lipids. We therefore hypothesized that flip-flop of negatively charged acidic lipids is the carrier of charge for the process of voltage-dependent fusion. The fact that voltage dependence could be eliminated by incorporating an acidic lipid into the membranes of either effector or target cells strongly indicates that the lipid acts locally at the site of hemifusion diaphragms where the two outer monolayers are merged. Fusion proteins accumulate at sites of diaphragms [39]. We found that the extents of fusion mediated by influenza HA, in contrast to VSV G, were not affected by incorporating NBD-PG into either the effector or target cell (Fig. 6). This leads us to conclude that the acidic lipid composition of the diaphragm–in either amount or species of lipid–is not critical for fusion.

We have previously shown that the voltage-dependent steps are downstream of hemifusion [5], and we have now found that the effects of NBD-PG are downstream as well. Because positive voltages should deplete acidic lipids from outer leaflets, this voltage should reduce the likelihood that in the merged outer leaflets, acidic lipids could interact with the TMD of the fusion protein. Combining all results leads us to propose that binding of acidic lipids to the portion of the TMD spanning the outer leaflet is essential for voltage-dependent fusion The critical feature that distinguishes TMDs of class I from those of class II and III in this respect is not yet clear.

Lipids are translocated between monolayers by flippases, floppases, and scramblases. The identities of flippases [40] and floppases [41] have been suggested, but uncertainty remains [42]. Anoctamin 6 (Ano6, the gene product of *TMEM16F*) is a calcium-activated and voltage-dependent anion channel [43], and relatively recently has been strongly associated with translocation of PS across plasma membranes [44], [45]. The voltage dependence of fusion could be due to voltage dependence of any of these transporters of PS, or to the charge movement carried by PS itself as it translocates between monolayers of a membrane. Although the molecular moieties that respond to voltage still need to be identified, the results of all our experiments lead to the conclusion that voltage dependence of fusion is a direct consequence of acidic lipid flip-flop.
